# Abbreviation definition identification based on automatic precision estimates

**DOI:** 10.1186/1471-2105-9-402

**Published:** 2008-09-25

**Authors:** Sunghwan Sohn, Donald C Comeau, Won Kim, W John Wilbur

**Affiliations:** 1 National Centre for Biotechnology Information, National Library of Medicine, National Institutes of Health, Bethesda, MD, USA

## Abstract

**Background:**

The rapid growth of biomedical literature presents challenges for automatic text processing, and one of the challenges is abbreviation identification. The presence of unrecognized abbreviations in text hinders indexing algorithms and adversely affects information retrieval and extraction. Automatic abbreviation definition identification can help resolve these issues. However, abbreviations and their definitions identified by an automatic process are of uncertain validity. Due to the size of databases such as MEDLINE only a small fraction of abbreviation-definition pairs can be examined manually. An automatic way to estimate the accuracy of abbreviation-definition pairs extracted from text is needed. In this paper we propose an abbreviation definition identification algorithm that employs a variety of strategies to identify the most probable abbreviation definition. In addition our algorithm produces an accuracy estimate, pseudo-precision, for each strategy without using a human-judged gold standard. The pseudo-precisions determine the order in which the algorithm applies the strategies in seeking to identify the definition of an abbreviation.

**Results:**

On the Medstract corpus our algorithm produced 97% precision and 85% recall which is higher than previously reported results. We also annotated 1250 randomly selected MEDLINE records as a gold standard. On this set we achieved 96.5% precision and 83.2% recall. This compares favourably with the well known Schwartz and Hearst algorithm.

**Conclusion:**

We developed an algorithm for abbreviation identification that uses a variety of strategies to identify the most probable definition for an abbreviation and also produces an estimated accuracy of the result. This process is purely automatic.

## Background

Abbreviations are widely used in biomedical text. The amount of biomedical text is growing faster than ever. In early 2007, MEDLINE included about 17 million references. For common technical terms in biomedical text, people tend to use an abbreviation rather than using the full term [1,2]. In this paper we interchangeably use the term *short form *(SF) for an abbreviation and *long form *(LF) for its definition. Along with the growing volume of biomedical texts the number of resulting SF-LF pairs will also increase. The presence of unrecognized words in text affects information retrieval and information extraction in the biomedical domain [3-5]. This creates the continual need to keep up with new information, such as new SF-LF pairs. A robust method to identify the SFs and their corresponding LFs within the same article can resolve the meaning of the SF later in the article. In addition, an automatic method enables one to construct an abbreviation and definition database from a large data set.

Another challenging issue is how to evaluate the pairs found by an automatic abbreviation identification algorithm, especially when dealing with a large and growing database such as MEDLINE. It is impractical to manually annotate the whole database to evaluate the accuracy of pairs found by the algorithm. An automatic way to estimate the accuracy of extracted SF-LF pairs is helpful to save human labor and to accomplish a full automatic processing of abbreviation identification and evaluation.

In this paper we propose an abbreviation identification algorithm that employs a number of rules to extract potential SF-LF pairs and a variety of strategies to identify the most probable LFs. The reliability of a strategy can be estimated which we term pseudo-precision (P-precision). Multiple strategies – each performing a specific string match – are applied sequentially, from the most reliable to the least reliable, until a LF is found for a given SF or the list is exhausted. Since the algorithm starts from the most reliable strategy it can identify the most probable LF if multiple LF candidates exist. No gold standard is required.

Many methods have been proposed to automatically identify abbreviations. Schwartz and Hearst [6] developed a simple and fast algorithm that searches backwards from the end of both potential SF and LF and finds the shortest LF that matches a SF. A character in a SF can match at any point in a potential LF, but the first character of a SF must match the initial character of the first word in a LF. They achieved 96% precision and 82% recall on the Medstract corpus [7] which was higher than previous studies [7,8]. Schwartz and Hearst also annotated 1000 MEDLINE abstracts randomly selected from the output of the query term "yeast" and achieved 95% precision and 82% recall. Their algorithm is efficient and produces relatively high precision and recall.

Yu et al. [9] developed pattern-matching rules to map SFs to their LFs in biomedical articles. Their algorithm extracts all potential LFs that begin with the first letter of the SF and iteratively applies a set of pattern-matching rules on the potential LFs from the shortest to longest until a LF is found. The pattern-matching rules are applied sequentially in pre-defined order. They achieved an average 95% precision and 70% recall on a small set of biomedical articles. They also manually examined whether 60 undefined SFs in biomedical text could be identified in four public abbreviation databases and found that 68% of them existed in these databases. Park and Byrd [10] also used a pattern-based method and achieved 98% precision and 95% recall on a small data set. They restricted SFs to character strings that start with alphanumeric characters, have a length between 2 and 10 characters, and contain at least one upper-case letter.

Chang et al. [8] used dynamic programming to align SFs with their LF. They computed feature vectors from the results of the alignment and used logistic regression on these features to compute the alignment score. They achieved 80% precision and 83% recall on the Medstract corpus [7]. Their algorithm provided probabilities (alignment scores) for the SF-LF pairs found by the algorithm.

An automatic method of abbreviation identification has also been developed for matching protein names and their abbreviations (Yoshida et al. [11]). They used the method of Fukuda et al. [12] to identify protein names in 23,469 articles published in March and July 1996 in MEDLINE and assumed that these protein names were correct. Then, they developed a set of rules to map protein names to their abbreviations and achieved 98% precision and 96% recall. This performance does not represent the actual precision and recall because they assumed that the automatically extracted protein names were all correct.

Our approach is similar to Yu et al. [9] in that we use multiple rules sequentially for mapping SFs to LFs until the LF is identified. Yu et al. tried to find the shortest LF candidate by iteratively applying their five rules on all potential SF-LF pairs. However we used relaxed length restrictions and tried to find the best LF candidate by searching for the most reliable successful strategy out of seventeen strategies. One of the major advantages of our algorithm is that the P-precision provides an estimate of the reliability of the identified SF-LF pairs. Thus, our algorithm rates the identified SF-LF pairs without any human judgment. This provides a confidence estimate for applications.

## Methods

### Data preparation

#### Potential SF and LF pairs

MEDLINE is a collection of bibliographic records pointing to the biomedical literature. All records have titles and about half have abstracts. Approximately 12 million potential SF-LF pairs were extracted from MEDLINE. Potential SFs are one or two words within parentheses and are limited to at most ten characters in total length. For our purpose white space and punctuation marks delineate word boundaries. We include single alphabetic characters as potential SFs because such abbreviations occur frequently in MEDLINE. Sequence or list indicators (e.g., (a) (b) (c), (i) (ii) (iii),...) and common strings ("see", "e.g.", "and", "comment", "letter", "author's transl", "proceeding", "=", "p <",...) were identified and not extracted as potential SFs (see Example 1). A potential SF must begin with an alphanumeric character and contain at least one alphabetic character. A potential LF consists of up to ten consecutive words preceding a potential SF in the same sentence (see Example 2). We used the sentence segmenting function in MedPost [13].

#### Example 1. Sequence or list indicators and common strings

Expression includes three components: (a) an increase of synaptic currents, (b) an increase of intrinsic excitability in GrC, and (c) an increase of intrinsic excitability in mf terminals. Based on quantal analysis, the EPSC increase is mostly explained by enhanced neurotransmitter release.

Here 'a', 'b', and 'c' are not extracted as potential SFs.

The major changes have been the recognition of the importance of dominant blood vessel size, the distinction between primary and secondary vasculitis and the incorporation of pathogenetic markers such as ANCA (see Table 6).

We recommend that the appropriate use of those top 10 statistics be emphasized in undergraduate nursing education and that the nursing profession continue to advocate for the use of methods (e.g., power analysis, odds ratio) that may contribute to the advancement of nursing research.

The mean lesion contrasted-to-noise ratio was significantly higher on the T1-weighted images (p < 0.05).

Here "see", "e.g.", and "p < 0.05" are not extracted as potential SFs.

#### Example 2. Potential SF-LF pairs

Comparison of two timed artificial insemination (TAI) protocols for management of first insemination postpartum.

Potential SF: TAI

Potential LF: Comparison of two timed artificial insemination

(The potential LF extends up to the beginning of the sentence.)

The higher the O(2) concentration the faster is the development of atelectasis, an important cause of impaired pulmonary gas exchange during general anesthesia (GA).

Potential SF: GA

Potential LF: important cause of impaired pulmonary gas exchange during general anesthesia

(The potential LF is up to ten consecutive words preceding a potential SF.)

### Strategies

The most common case of a SF is an acronym in which each character of the SF matches the first character of a word in the LF. However, many SFs do not follow this rule. There are many variations. A character of a SF may match any character within a word of a LF, not just the first character. Also a character in a SF may not match any character in the LF. Some words in a LF may be skipped and not contain a match to any character in the SF. In order to identify SFs and their corresponding LFs reliably, numerous strategies that deal with possible matching patterns are necessary. For this reason we developed a variety of strategies, of varying reliability, which cover most matching patterns in biomedical text. First, we implemented the most common and reliable strategy people use to identify an acronym SF. Then, we implemented the next most common strategy on the remaining potential SF-LF pairs that were missed by the previous strategy. We kept adding new strategies until we had covered the most common strategies used to construct abbreviations. We did not include all possible strategies as some would be quite complex in construction yet rare in occurrence.

For all our strategies, each character in a potential SF is matched to a particular character in a potential LF. All strategies also try to identify a LF by moving right to left and matching SF characters to characters within a potential LF in the same order. The idea of a backward search was also used in previous studies and worked effectively [6,11]. The first character in a SF must match either the initial character at the beginning of a LF or the first alphanumeric character following some non-alphanumeric character in the first token in a LF. Non-alphanumeric characters in a SF are skipped in the matching process. The LF found by a strategy must also pass additional checks. The LF is only considered valid if the number of characters in the LF is greater than that of the SF and the LF does not contain the SF as a space delimited substring. Table 1 shows basic rules applied in our strategies and Table 2 provides detailed explanation and examples of each strategy.

**Table 1:** Basic rules used in strategies

**Rule**	**Example**
**FL**: A letter of SF matches the 1st letter of a word^a ^in LF.	**L**etter
**FC**: A character of SF matches the 1st character of a word in LF.	**1**-word
**FCG**: A character of SF matches the character following a non-alphanumeric non-space character in LF.	Word-**W**ord
**LS**: The last character of SF is 's' and matches the last character of LF 's'.	Word**s**
**NF**: A character of SF matches any character except the 1st character in LF.	W**o**rd, Wo**r**d, Wor**d**
**SBW**: A character of SF matches a character within a word in LF and the substring of that LF word from the match until the end of the word is a defined word^b^.	Word**W**ord
**CL**: A substring of SF matches any two or more consecutive characters of a word in LF.	**Wo**rd, W**or**d, Wo**rd**
**ST**: While matching SF with LF, skip a stopword in LF.	Stopword
**SK**: While matching SF with LF, skip a word in LF.	Word
**AC**: A character of SF matches any character in LF	**W**ord, W**o**rd, Wo**r**d, Wor**d**

^a ^Words are white space demarcated strings. This applies to all rules.

^b ^A defined word is at least three letters, a non-stopword, and appears at least 100 times in MEDLINE.

**Table 2:** Strategy description

**Strategy**	**Example**
FirstLet: FL for all letters in SF	**A**lpha **B**eta (AB) Fail: Alpha-Beta (AB)
FirstLetOneChSF: Applied for 1-letter SF. FL with restrictions^a^.	**D**opamine (D)
FirstLetGen: FC or FCG, at least one FCG	1-**A**lpha-**B**eta (AB), **A**lpha-**B**eta (AB) Fail: Alpha Beta (AB)
FirstLetGen2: FC or FCG	**A**lpha **B**eta (AB), **A**lpha-**B**eta (AB)
FirstLetGenS: SF consists of upper-case letters and lower-case letter 's' at the end. LS for final 's' in SF and FC for the rest	**A**lpha **B**eta**s **(ABs) Fail: Alpha Beta Gammas (ABs)
FirstLetGenStp: FC or FCG or ST, at least one ST (at most one ST between matched words or at end)	**A**lpha and **B**eta (AB) Fail: Alpha Beta (AB), Alpha word Beta (AB)
FirstLetGenStp2: FC or FCG or ST, at least one pair of adjacent ST (at most two ST between matched words or at end)	**A**lpha of the **B**eta (AB) Fail: Alpha Beta (AB), Alpha and Beta (AB)
FirstLetGenSkp: FC or FCG or SK, at least one SK (at most one SK between matched words or at end)	**A**lpha and **B**eta (AB), **A**lpha word **B**eta (AB) Fail: Alpha Beta (AB)
WithinWrdFWrd: FC or FCG or SBW, at least one SBW, all SBW in a FC or FCG matched word in LF	**A**lpha**B**eta (AB) **A**lpha **B**eta**G**amma (ABG) Fail: **A**lpha**B**eta in**G**amma (ABG) (SBW but no FC in inGamma)
WithinWrdWrd: FC or FCG or SBW, at least one SBW	**A**lpha**B**eta (AB), **A**lpha**B**eta in**G**amma (ABG) Fail: AlphaBxx (AB) (Bxx is not defined-word) Alpha Beta (AB) (no SBW)
WithinWrdFWrdSkp: WithinWrdFWrd or SK, at least one SK (at most one SK between matched words or at end)	**A**lpha**B**eta word **G**amma (ABG) Fail: AlphaBeta Gamma (ABG)
WithinWrdFLet: FC or FCG or NF, at least one NF, all NF in a FC or FCG matched word in LF	**A**lpha**B**xx (AB) Fail: AlphaBxx inCxx (ABC), Alpha Bxx (AB)
WithinWrdLet: FC or FCG or NF, at least one NF	**A**lpha**B**xx (AB), **A**lpha**B**xx in**C**xx (ABC) Fail: Alpha Bxx(AB) (no NF)
WithinWrdFLetSkp: WithinWrdFLet or SK, at least one SK (at most one SK between matched words or at end)	**A**lpha**B**xx word **G**amma (ABG) Fail: AlphaBxx Gamma (ABG)
ContLet: FC or FCG or CL, at least one CL, all CL in a FC or FCG matched word in LF	**AB**xx (AB), **AB**xx **C**xx (ABC), **A**xx**BC**xx (ABC) Fail: ABxx xCxx (ABC), xABxx (AB)
ContLetSkp: ContLet or SK, at least one SK (at most one SK between matched words or at end)	**AB**xx and **C**xx (ABC), **AB**xx word **C**xx (ABC) Fail: ABxx Cxx (ABC)
AnyLet: The 1st character of SF: FC or FCG. The others: AC or SK (at most one SK between matched words or at end)	**A**lpha x**B**eta (AB), **A**lpha word x**B**eta (AB)

^a ^The SF and LF pair must appear in MEDLINE at least twice and LF must not be a stopword.

### Pseudo-precision

For each strategy that we use, we estimate its accuracy by what we term a pseudo-precision. The basic idea is that we try a strategy to match a given SF on potential LFs for which we know it is not the correct SF. The rate at which this produces matches is then our estimate of the tendency to produce erroneous matches with that SF and that strategy. We then discount the matches we find on potential LFs which are paired with that SF at that same rate. What remains are what we count as correct and the resulting fraction of all matches is our estimated pseudo-precision.

To be more formal consider a particular set of potential SF-LF pairs *X *(for details, see the way of grouping SF-LF pairs of MEDLINE in the next part "Assigning P-precision to a Strategy"). Label the unique potential SFs *s*_*t *_(*t *= 1,.., *m*; *m *is the number of unique SFs in the set). Let *X*_*S*_(*s*_*t*_) be the subset of *S *that has potential SF *s*_*t *_and *X*_*L*_(*s*_*t*_, *A*) be the subset of *X *that satisfies strategy *A *using *s*_*t*_. (see Example 3).

#### Example 3. Examples of X_L_(s_t_, A) set

The list of examples are all retrieved using the strategy FirstLet with short form "CAT" or short form "LBA" (formatted as potential-SF|potential-LF, the bold denotes matches).

*X*_*L *_("*CAT*", *FirstLet*)

   ATP|material was a mixture of the adenyl **c**ompounds **a**denosine **t**riphosphate

   CAT|routine examination of the posterior fossa by **c**omputer **a**ssisted **t**omography†

   CAT|**C**omputerised **a**xial **t**omography†

   LBA|In Part I of this **c**ommunication, **a t**echnique

   TFP|NDGA); anti-oxidant, vitamin E; and **c**almodulin **a**ntagonists, **t**rifluoperazine

   TSH|) and triiodothyronine (T3) serum **c**oncentrations, **a**nd **t**hyrotropin

*X*_*L *_("*LBA*", *FirstLet*)

   BFA|Since the fungal **l**actone **B**refeldin **A**

   BGA|During the remission course of ISs, **l**ow-voltage **b**ackground **a**ctivity

   BKA|The ANT **l**igands **b**ongkregkic **a**cid

   LBA|and manufacturing techniques are known from the **L**ate **B**ronze **A**ge‡

   LBA|were compared to its prototype predecessor assay, **L**ine **B**lot **A**ssay‡

   USA|HLA genes of Aleutian Islanders **l**iving **b**etween **A**laska

In Example 3 the list under *X*_*L *_("*CAT*", *FirstLet*) are potential SF-LF pairs that satisfy the FirstLet strategy using SF "CAT". Note that the actual SF can be different from "CAT". In the pairs whose SF is not "CAT", the identified LFs by FirstLet are incorrect. The correct LFs can be identified by using a different strategy in some cases (*ATP|adenosine triphosphate; TFP| trifluoperazine*). The SF TSH abbreviates a synonym for *thyrotropin*. The pairs labelled with '†' at the end are elements in the set *X*_*S *_("*CAT*") ∩ *X*_*L *_("*CAT*", *FirstLet*). Similarly the list under *X*_*L *_("*LBA*", *FirstLet*) are potential SF-LF pairs that satisfy the FirstLet strategy using SF "LBA". Like the previous examples there is a false SF ("USA") and some LFs can be correctly identified by using a different strategy than FirstLet (*BGA|background activity; BFA|Brefeldin A; BKA|bongkregkic acid*). The pairs labelled with '‡' at the end are elements in the set *X*_*S *_("*LBA*") ∩ *X*_*L *_("*LBA*", *FirstLet*).

Let us denote the size of sets by

(1) *N *= ||*X*||

(2) *n*_*S *_(*s*_*t*_) = ||*X*_*S *_(*s*_*t*_)||

(3) *n*_*L *_(*s*_*t*_, *A*) = ||*X*_*L *_(*s*_*t*_, *A*)||.

Also, define the size of the intersection of *X*_*S *_(*s*_*t*_) and *X*_L _(*s*_*t*_, *A*) as

(4) *n*_*SL *_(*s*_*t*_, *A*) = ||*X*_*S *_(*s*_*t*_) ∩ *X*_*L *_(*s*_*t*_, *A*)||.

Define *λ *by

(5) $$\lambda =\frac{\left\|X_{L}\left(s_{t},A\right)\cap \left(X-X_{S}\left(s_{t}\right)\right)\right\|}{\left\|X-X_{S}\left(s_{t}\right)\right\|}.$$

Here *λ *is the rate of success for strategy *A *using *s*_*t *_on the pairs whose SF is not *s*_*t*_. The denominator is the number of pairs where the SF is not *s*_*t *_and the numerator is the number of those pairs where strategy *A *using *s*_*t *_succeeded anyway. Thus *λ *represents the chance rate of success for the strategy without regard to the paired SF. Then, we define the P-precision of strategy *A *for SF *s*_*t*_

(6) $$prec_{A}\left(s_{t}\right)=\frac{n_{SL}\left(s_{t},A\right)-\lambda n_{S}\left(s_{t}\right)}{n_{SL}\left(s_{t},A\right)},$$

The value *λn*_*S *_(*s*_*t*_) in equation (6) is our estimate of the number of pairs that have SF *s*_*t *_and satisfy strategy *A *using *s*_*t *_merely by chance. Thus when we remove this portion from *n*_*SL *_(*s*_*t*_, *A*) the value of the numerator in equation (6) becomes significant or meaningful matches using strategy *A*. This value is divided by *n*_*SL *_(*s*_*t*_, *A*), which is the observed number of successes of the strategy, and so the P-precision becomes the estimated success rate (accuracy) of strategy *A *using *s*_*t*_. The analogy is with the expression for precision

(7) $$\text{Precision}=\frac{\text{AllPositives}-\text{FalsePositives}}{\text{AllPositives}}.$$

The P-precision *prec*_*A *_(*s*_*t*_) is based on a statistical notion of observing the occurrence of a potential SF-LF match at a rate above chance occurrence. If a strategy matches a SF to its potential LF and this match was not produced by chance and if the strategy is a reasonable one that one might very well use to produce an abbreviation, then it is likely that this strategy was actually used to produce this SF. On this basis we have concluded that P-precision is a useful approximation to true precision. The more reliable the strategy the higher the actual satisfaction (*n*_*SL *_(*s*_*t*_, *A*)) is compared to the expected chance satisfaction (*λn*_*S *_(*s*_*t*_)). Hence, the more reliable the strategy, the higher the P-precision. The P-precision of strategy *A *for a given set is the weighted average over all SFs in the set,

(8) $$prec_{A}=\frac{\sum_{t=1}^{m}prec_{A}\left(s_{t}\right)n_{SL}\left(s_{t},A\right)}{\sum_{t=1}^{m}n_{SL}\left(s_{t},A\right)}.$$

### Assigning P-precision to a strategy

We developed various strategies (see Table 2) and each involves a different type of pattern matching to identify a LF. Some strategies are more reliable for defining LFs and some are less reliable. Thus, assigning higher priority to a more reliable strategy is necessary to determine the best candidate LF if multiple LF candidates exist. Reliability of a strategy can be different for different types of SFs. For this reason, we divided all potential SF-LF pairs obtained from MEDLINE into six groups based on the number of characters in the SF: 1, 2, 3, 4, 5, and 6+. Each group, except 1-letter SF, was further divided into three sub-groups: SFs consisting of all alphabetic characters, at least one digit plus alphabetic characters, and at least one non-alphanumeric character. For each group we evaluated strategies and ordered them based on their P-precision. The SF group 6+ used the same strategies as the 5-character SF group.

**Figure 1:**
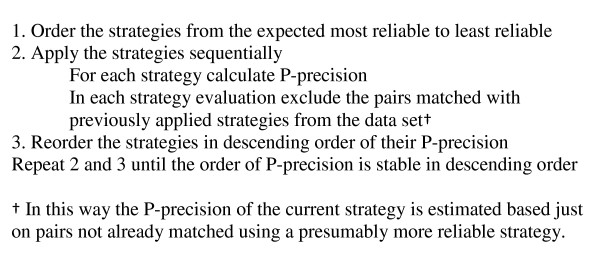
Strategy ordering.

The order (priority) of strategies is in descending order of the P-precision – from the most reliable to the least reliable strategies. We evaluate the reliability of our strategies by their P-precision, equation (8). For each group of potential SF-LF pairs we ordered the list of strategies. Figure 1 shows the detailed process. This process allowed us to determine the best ordering of strategies for each group based on their P-precisions.

### Application

Our process of abbreviation identification in free text consists of 1) extracting potential SF-LF pairs, 2) for each potential SF-LF pair applying the strategies corresponding to the given SF group, and 3) identifying the most reliable SF-LF pair. Each SF group has its own prioritized strategies with their corresponding P-precisions specific to that group. The strategies are applied sequentially in predefined order and the process stops with the first strategy that succeeds. In this way we can find the most reliable LF if more than one possible LF exists. The algorithm identifies a SF-LF pair and assigns the P-precision of the strategy that found it.

To increase recall of our algorithm we look at potential SF-LF pairs associated with square brackets in addition to parentheses. Also, we consider both "LF (SF)" and "SF (LF)" orders. When we consider the "SF (LF)" order a potential SF is one word containing at least one upper-case letter. If both "LF (SF)" and "SF (LF)" cases are successful we choose the one with the higher P-precision. Because a SF must consist of at most ten alphabetic characters, if the text inside parentheses or square brackets contains ';' or ',' we treat the text before these punctuation marks as a potential SF (e.g., alpha beta (AB, see reference) – "AB" is extracted as a potential SF). This also increases the number of potential SF-LF pairs and has a positive effect on recall.

### Evaluation

For our definitive evaluation we annotated 1250 records, which have both title and abstract. These were randomly selected from MEDLINE. The four authors individually annotated 250 records each. The backgrounds of the four are: medical science, chemistry, information science, and computer engineering. An additional 250 records were annotated by all four authors in order to test inter-annotator agreement. After initial annotation we checked the pairs that were identified by either our algorithm or the Schwartz and Hearst algorithm but were not in the gold standard. All four annotators consulted together regarding these pairs and added to the gold standard those judged correct.

We compared each annotator's performance on the 250 records judged in common. Two pairs of annotators worked to create two reconciled versions. Then all four annotators worked together to make final judgments where there was disagreement. The result includes 237 SF-LF pairs. This consensus was used to rate each annotator's work and the two pairwise reconciled versions (Table 3). Three annotators' results were similar and one annotator's result was somewhat lower than the other three. However, both pairwise reconciliations were closer to the consensus than any single annotator's work.

**Table 3:** Difference between gold standard and annotators in 250 MEDLINE records.

Annotator	Precision (%)	Recall (%)	F-measure^a ^(%)
Annotator 1	93.4	89.5	91.4
Annotator 2	95.6	91.6	93.5
Annotator 3	93.4	89.5	91.4
Annotator 4	89.7	73.4	80.7
Annotator 1 & 2	96.1	92.8	94.4
Annotator 3 & 4	98.7	96.2	97.4

^a ^F-measure = 2*(Precision*Recall)/(Precision+Recall)

## Results

We tested our algorithm on the Medstract corpus [7] which has been used in previous studies [6-8]. The gold standard of Medstract has 168 SF-LF pairs. We annotated this data set manually since only the text is available to the public. Note that the gold standard for other studies might be slightly different. Our algorithm produced 97% precision and 85% recall. For comparison Schwartz and Hearst achieved 96% precision and 82% recall (These precision and recall figures were reported in the Schwartz and Hearst's paper. On our annotated version of Medstract their algorithm produced 96% precision and 83% recall.), Chang et al. achieved 80% precision and 83% recall, and Pustejovsky et al. achieved 98% precision and 72% recall. Most pairs missed by our algorithm are ones with unmatched characters in the SF (e.g., *Fob1|fork blocking, 5-HT|serotonin*), out of order match (e.g., *TH|helper T*), and partial match (e.g., *cAMP|3',5' cyclic adenosine monophosphate*).

The gold standard of 1250 MEDLINE records includes 1221 true SF-LF pairs. Our algorithm identified 1053 pairs with 1016 correct pairs – 96.5% precision and 83.2% recall. Table 4 shows some examples of correctly identified SF-LF pairs along with the P-precision and strategy used. Most correct cases were assigned high P-precision except for "GC/ECD" that was identified by the AnyLet strategy. False positive (FP) pairs with high P-precision were unusual. The SF "*IVA-SIV*" was matched to "*Ivanovas-Sieve colony*" with 0.99 P-precision. This pair was annotated as a synonym pair but not considered an abbreviation in our gold standard. The SF "*pHo*" was identified as the LF of "*pH*" with 0.96 P-precision from the phrase, "... *extracellular pH (pHo)*...". The true SF-LF pair is "*pHo*|*extracellular pH*" in which the character 'o' in SF does not match any character in the LF. Generally, FP cases were assigned relatively low P-precision (e.g. (formatted by SF|LF|P-precision), *apathy|acquired knowledge, important changes of personality|0.74, CL-EE|cellulose hollow fiber dialyzer|0.86*).

**Table 4:** Correct SF and LF pairs identified by our algorithm.

SF	LF	P-precision	Strategy Used
IBV	infectious bronchitis virus	0.9998	FirstLet
CZE	capillary zone electrophoresis	0.9998	FirstLet
PMECs	pulmonary microvascular endothelial cells	0.9999	FirstLetGenS
LCM	Lymphocytic choriomeningitis	0.9978	WithinWrdFWrd
ICG	impedance cardiogram	0.9978	WithinWrdFWrd
D-Gal	D-Galactosamine	0.9946	ContLet
Prl	prolactin	0.9877	ContLet
P	progesterone	0.9672	FirstLetOneChSF
T	Testosterone	0.9672	FirstLetOneChSF
SKY	spectral karyotyping	0.9813	WithinWrdFLet
GG	genioglossus	0.9420	WithinWrdFLet
TEV	tobacco etch potyvirus	0.9437	WithinWrdWrd
PDX1	pancreatic duodenal homeobox factor-1	0.9863	WithinWrdLet
GC/ECD	gas chromatography employing an electron capture detector	0.7456	AnyLet

Pairs missed by our algorithm demonstrate strategies not included in our list of seventeen: pairs with unused characters in the SF (e.g., *K|control, bNOS|neuronal NO synthase*), out of order match (e.g., *DM|Myotonic dystrophy*), mapping digits in a SF to words in a LF (e.g., *3D|three-dimensional*), and conjunction (e.g., *DEHP|di-2-ethylhexyl-phthalate, DnOP| di-n-octyl phthalate*, from the phrase "...di-2-ethylhexyl-[DEHP] and di-n-octyl-[DnOP] phthalate..."). Our algorithm does not allow LFs to skip more than one non-stopword between words to avoid inappropriate LF candidates. Some SF-LF pairs require skipping more than one non-stopword between words in the LF and our algorithm fails for those pairs (e.g., *COMMIT|Community Intervention Trial for Smoking Cessation, FHPD|family history method for DSM-III anxiety and personality disorders*).

The gold standard includes 23 1-letter SFs. Our algorithm achieved 100% precision and 83% recall on 1-letter SFs. It missed four cases. For one of them the LF consists of two words, which our algorithm does not recognize (i.e., *R|respiratory quotient*).

Among our strategies AnyLet is the least reliable strategy and the last option to be tried. It is of interest to apply the algorithm without the AnyLet strategy. The resulting algorithm achieves 96.9% precision and 83.1% recall. The recall is close to the original algorithm (83.2%) and precision is a little higher than the original algorithm (96.5%).

**Figure 2:**
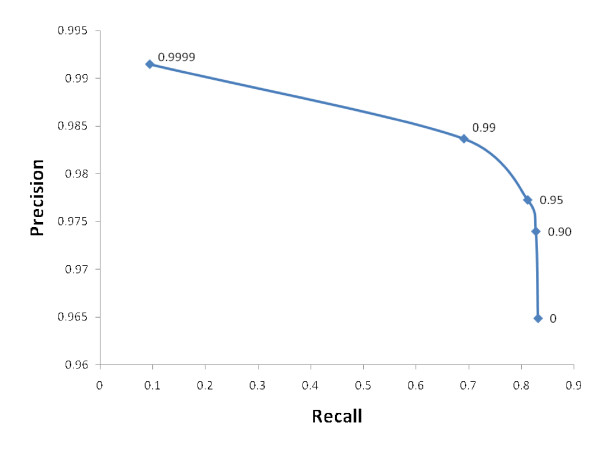
**Precision-recall curve with P-precision threshold on 1250 MEDLINE records**. Some values of P-precision are labelled on the curve.

This can be extended to any precision threshold. Figure 2 shows the precision-recall curve on the 1250 MEDLINE records. The precision and recall were calculated with different threshold values of P-precision. For example, with the threshold of 0.90 P-precision we retrieved the identified SF-LF pairs by the algorithm only if their P-precision is greater than 0.90. With P-precision threshold 0.9999 the algorithm produced 99.2% precision at 10% recall, with 0.99 threshold 98.4% precision at 69.1% recall, and with 0.90 threshold 97.4% precision at 82.8% recall. This result shows that the pairs identified with high P-precision are more likely to be true positive (TP).

**Figure 3:**
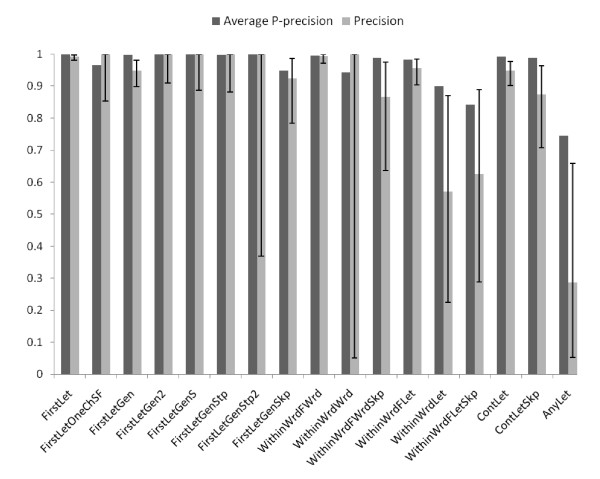
**Evaluation of strategies on 1250 MEDLINE records**. Average P-precision is the mean of P-precisions of SF-LF pairs identified by that strategy (dark bars) and Precision is the number of gold standard pairs identified by a given strategy divided by the number of pairs identified by that strategy (light bars). An error bar denotes the 95% confidence interval of Precision. Long error bars correspond to small sample size for that strategy.

Using our 1250 MEDLINE record gold standard we examined the correlation between P-precision and true precision (Figure 3). The average P-precision of most strategies lies within the 95% confidence interval of Precision or a little higher than the upper limit of Precision. This suggests that our P-precision is a reasonable estimate of a strategy's actual precision, though it may be at times a mild overestimate.

## Discussion

In a previous study of automatic abbreviation identification Schwartz and Hearst [6] developed a simple and fast algorithm that performed better or at least as well as previous methods. We compared the performance of our algorithm with theirs on the same 1250 MEDLINE records used in our evaluation. Schwartz and Hearst found 1013 pairs with 957 correct pairs – 94.5% precision and 78.4% recall. We have 2% and 4.8% higher precision and recall, respectively. The major differences between our approach and Schwartz and Hearst's are: 1) we identify 1-letter SFs but Schwartz and Hearst do not identify them even though they included 1-letter SFs in the gold standard in their experiment; 2) we select highest P-precision LF if multiple LF candidates exist but Schwartz and Hearst select the shortest LF candidate (e.g., ours vs. Schwartz and Hearst: *IIEF|International Index of Erectile Function*, vs.*IIEF|Index of Erectile Function*, *PPIs|proton pump inhibitors *vs.*PPIs|pump inhibitors*); 3) we identify SF-LF pairs occurring within nested parentheses but Schwartz and Hearst give nested parentheses no special treatment; 4) Schwartz and Hearst allow more consecutive skipped words without matching. This can result in success (*COMMIT|Community Intervention Trial for Smoking Cessation*) or failure (*that is|trials in which patients were assigned to a treatment group; range|RESULTS: The median patient age at diagnosis was 7.5 years; SPEMs|schizophrenia: a preliminary investigation of the presence of eye-tracking*); 5) occasionally, the Schwartz and Hearst restriction on LF length (min(|SF|+5, |SF|*2)) causes failure on LFs including many stopwords (*CHP|carcinoma of the head of the pancreas; QOF|questionnaire on the opinions of the family*. Our strategy, FirstLetGenStp2 can identify these cases.

Our algorithm uses a variety of strategies to identify the SF-LF pairs. Those strategies are evaluated on different groups of SF-LF pairs in the MEDLINE database to estimate their reliability as P-precisions. The whole process of computing P-precisions on the total MEDLINE database and adjusting the ordering of strategies for each group required about two weeks on a high-end server (2 CPUs, 4GB of memory). We believe that the resulting algorithm would perform well on biological text from sources other than MEDLINE, such as full text journal articles. This opinion is based on the fact that it is largely the same authors that produce the text in MEDLINE that also produce journal articles. However, we have not carried out an evaluation on full text articles. However, for text from a subject area other than biology one might need to repeat the training process described in Figure 1.

Our algorithm took 25 seconds to process our 1250 MEDLINE record test set. Applied to the same set the Schwartz and Hearst algorithm took 0.38 seconds. While our algorithm is clearly not as fast, it is not so slow as to be a serious issue. Our algorithm can process all eighteen million MEDLINE records in about 2 and a half days.

## Conclusion

In this work we have developed a general approach which allows us to estimate the accuracy of a strategy for identifying an abbreviation, which we term P-precision. By gathering a number of strategies which provide a reasonably complete coverage of how authors actually construct abbreviations and computing their corresponding P-precisions we are able to construct an algorithm for abbreviation definition identification. The algorithm has the advantage that it is very competitive with existing algorithms in terms of accuracy and that it provides a P-precision estimate for each result it produces. Such estimates can be beneficial to applications which have stringent accuracy requirements or have accuracy requirements which vary. One could add additional strategies to our algorithm (though we would expect only a small gain in recall) or start with a completely different set of strategies and apply this same general approach.

One of the issues in automatic abbreviation identification is how to handle special cases that cannot be found by simple string matching, i.e., SFs containing characters that do not appear in the LF. Many pairs missed by our algorithm belong to this case. Interesting work on this problem has been done by Liu and Friedman [14] and Zhou et al. [15]. In future work we would like to find a way to use statistical evidence from multiple occurrences to not only find the matching SF-LF pairs but also make P-precision estimates for those pairs similar to the estimates we are currently making in the case where every letter from the SF is matched into the LF.

## Availability and requirements

Software implementing the algorithm presented here and files containing the 1250 annotated MEDLINE records are available for download at the project home page. At this site the algorithm is given the name AB3P (Abbreviation Plus P-Precision).

Project name: Abbreviations Plus Pseudo-Precision (Ab3P)

Project home page: 

Operating system(s): Unix (Linux)

Programming language: C++

License: United States government production, public-domain

## Authors' contributions

SS developed methods, implemented the software, and drafted the manuscript. DC and WK were involved in implementing the software and revising the manuscript. JW was responsible for all aspects of the project, contributed software, and helped revise the manuscript.
